# Automated identification of Monogeneans using digital image processing and K-nearest neighbour approaches

**DOI:** 10.1186/s12859-016-1376-z

**Published:** 2016-12-22

**Authors:** Elham Yousef Kalafi, Wooi Boon Tan, Christopher Town, Sarinder Kaur Dhillon

**Affiliations:** 1 Institute of Biological Sciences, Faculty of Science, University of Malaya, Kuala Lumpur, Malaysia; 2 Computer Laboratory, University of Cambridge, Cambridge, CB3 0FD UK

**Keywords:** Automated image recognition, Monogenean, K-nearest neighbour, Digital image processing

## Abstract

**Background:**

Monogeneans are flatworms (Platyhelminthes) that are primarily found on gills and skin of fishes. Monogenean parasites have attachment appendages at their haptoral regions that help them to move about the body surface and feed on skin and gill debris. Haptoral attachment organs consist of sclerotized hard parts such as hooks, anchors and marginal hooks. Monogenean species are differentiated based on their haptoral bars, anchors, marginal hooks, reproductive parts’ (male and female copulatory organs) morphological characters and soft anatomical parts. The complex structure of these diagnostic organs and also their overlapping in microscopic digital images are impediments for developing fully automated identification system for monogeneans (LNCS 7666:256-263, 2012), (ISDA; 457–462, 2011), (J Zoolog Syst Evol Res 52(2): 95–99. 2013;). In this study images of hard parts of the haptoral organs such as bars and anchors are used to develop a fully automated identification technique for monogenean species identification by implementing image processing techniques and machine learning methods.

**Result:**

Images of four monogenean species namely *Sinodiplectanotrema malayanus*, *Trianchoratus pahangensis*, *Metahaliotrema mizellei* and *Metahaliotrema* sp. (undescribed) were used to develop an automated technique for identification. K-nearest neighbour (KNN) was applied to classify the monogenean specimens based on the extracted features. 50% of the dataset was used for training and the other 50% was used as testing for system evaluation. Our approach demonstrated overall classification accuracy of 90%. In this study Leave One Out (LOO) cross validation is used for validation of our system and the accuracy is 91.25%.

**Conclusions:**

The methods presented in this study facilitate fast and accurate fully automated classification of monogeneans at the species level. In future studies more classes will be included in the model, the time to capture the monogenean images will be reduced and improvements in extraction and selection of features will be implemented.

## Background

Parasitic organisms have categorical homogeneous morphology, hence, pattern recognition techniques can be used to identify them [1]. The monogenean species are used in this study because they are worthy taxons for investigation [2]. There might be around 25000 species of monogenean in the world while barely 4000 of them are currently known [3]. Monogeneans are very diversified in terms of morphology and they are the only flatworm clade that have advanced adaptive radiation [2], with the variation of structural designs in the attachment organs [4], which are usually used for species identification. In particular, the haptoral attachment organ is characterized by sclerotized structures such as anchors, bars, hooks, etc. The morphology of these organs are usually unique to monogenean species [5] and are used as diagnostic characters in taxonomy [6, 7].

Automated classification of specimens’ images to their corresponding species requires development of models and methods that are able to characterize a specie’s morphology and apply this knowledge for their recognition. Automated systems should be combined with databases of images or text based information [8]. Primo Coltelli et al. [9] believe that image acquisition is the most important step in designing an automated system and capturing images should be well-focused with less complexity. The acquisition condition should be defined clearly and kept equal for all images, later labelled by expert taxonomists.

Automated systems may identify specimens at the species, genus, family or order levels using image processing, feature extraction and classification. Digital images of species, especially microscopic images, usually exhibit dust or other noise artefacts. Noise makes neighbouring pixel values cluster [10], so it should be reduced by image processing, in particular the smoothing methods of filtering. It is important to know the prevalent types of noise to be filtered so that it can be removed more efficiently. Besides this, the aim of image processing in the system is usually to transform digital images to a standard pose [11] and achieving recognizable objects on a uniform background, using segmentation. In order to facilitate the segmentation step, image artefacts should be removed and contrast as well as dynamic range has to be improved. The goal is to identify and classify objects of interest in digital images.

The performance of feature extraction and selection techniques depends on the type of a system’s classifiers and the quality of the data [12]. In order to achieve a high performance classification, not all features are required to be detected. If employed classifiers are strong enough, even if some features are left undetected, the method may yield successful results [13]. In KNN classification, objects are classified according to majority vote of their neighbours, with the objects being assigned to the class which is most common amongst its k nearest neighbours. In this classification method, objects which are close to each other according to their features, are likely to belong to the same pattern class [14]. The neighbours are taken from a set of training samples where the correct classification is known. For example in identification of species of Gyroactylus genus in fish ectoparasite [15], features that were extracted by Active Shape Models (ASM) were implemented to create two linear classifiers, Linear Discriminant Analysis (LDA) and K-nearest neighbor (KNN), and two non-linear classifiers, Multilayer Perceptron (MLP) and Support Vector Machine (SVM). KNN yielded the most ideal results with a classification accuracy of 98.75%.

Many semi-automated systems have been developed for identification of biological images in different levels. In 1996, the Dinoflagellate categorization (DiCANN) system, based on neural networks [16] was developed. Subsequently, forensic identification of mammals according to their single hair patterns under a microscope was investigated by Moyo et al. [17]. Yuan et al. [18] discussed the identification of rats up to the species level from images of their tracks. Later the improvements in semi-automated systems resulted in fully automated systems. Examples of successful existing automated systems are the automated Leafhopper Identification system (ALIS) [19], the Digital Automated Identification System (DAISY) [20], the Automatic Identification and characterization of Microbial Populations (AIMS) [21], the Automated Bee Identification System (ABIS) [22], BugVisux [23], automated identification of bacteria by use of statistical methods [10], an automated identification system which estimates whiteflies, aphids and thrips densities in a greenhouse [24], Species Identification, automated (SPIDA) [25], But2fly [26], Automated Insect Identification through Concatenated Histograms of Local Appearance (AIICHLA) [13], an automated identification system for algae [9] and Automated identification of copepods [27]. In the work done by Arpah et al. (2013) [28], illustration images of haptoral bar of monogeneans were used in building a content based image retrieval system. Contrary to the previous study, in thecurrent study, we digitised microscopic specimen images to develop a monogenean species identification technique.

## Methods

**Fig. 1:**
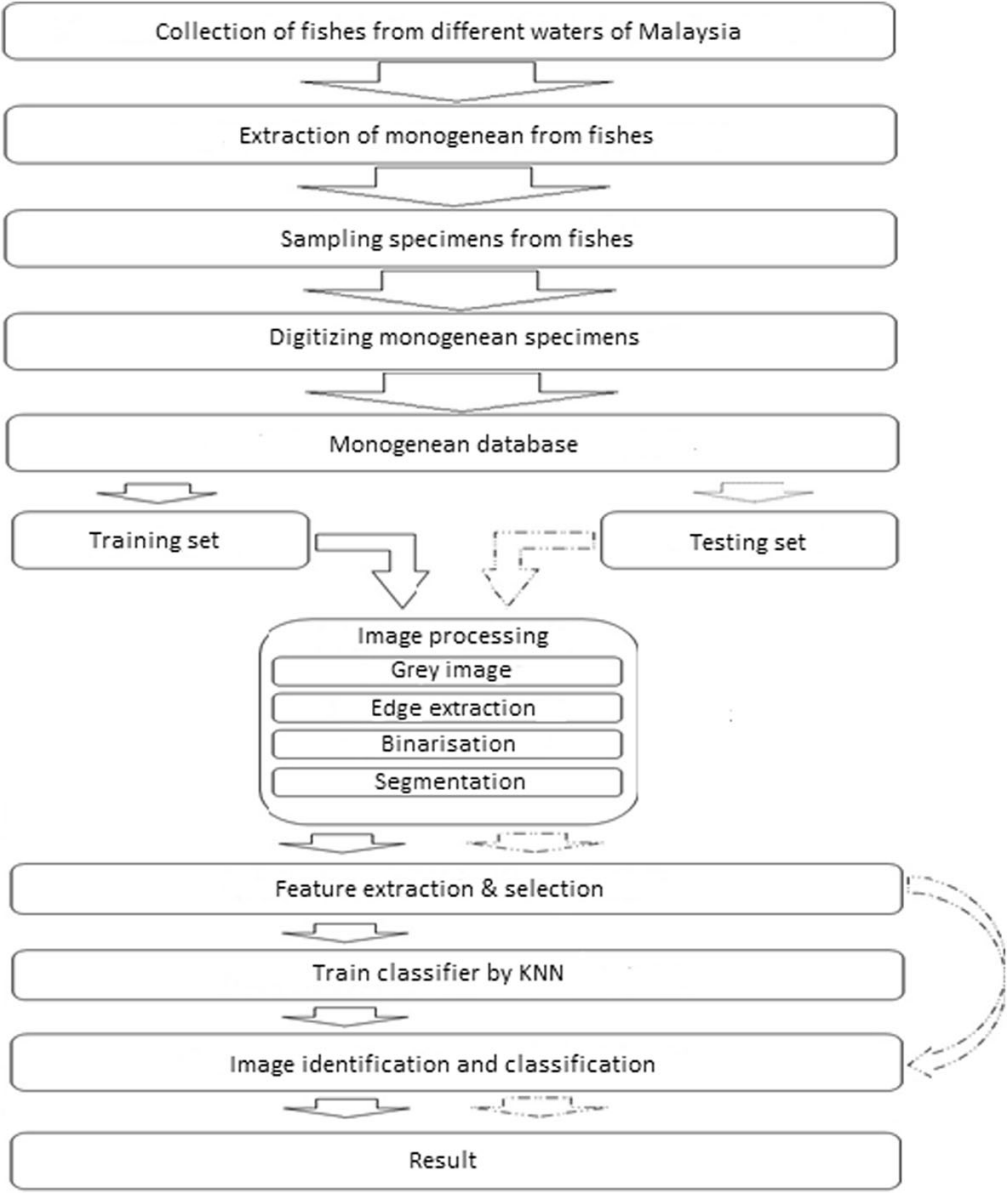
System flowchart

The study’s approach followed the methodology which is detailed as follows. Figure 1 shows the system workflow.

### Data collection

Monogeneans were collected from gills of killed or captured specimens. The attached tissues were removed using fine needles and placed on clean slides with a drop of water under a coverslip. Later the water was removed and four corners of coverslip were fixed. Subsequently, specimens were cleared using Ammonium pirate glycerine. Digital images of the hard anatomical structures of the monogeneans were taken using a Leica digital camera attached to Leica microscope at 40× magnification. Recognition of monogeneans is based on shape and size of their hard parts which are dorsal and ventral anchors, bars, as well as their male and female compulatory organs [29]. Thus, accurate recognition of monogeneans is very much dependant on features which are extracted from these parts. Our database consist of 23 species’ images, however in this study we randomly picked 4 species : *Sinodiplectanotrema malayanus*, *Trianchoratus pahangensis*, *Metahaliotrema mizellei* and *Metahaliotrema* sp. (undescribed).

The modular image processing and analysis software package used is QWin Plus, due to its versatile architecture, designed to solve demanding quantitative analysis tasks. MATLAB R2013a was used to pre-process and extract features from monogenean digital images.

### Preparation of slides of monogenean specimens

The slides of monogeneans used in this study were collected by experts since 1996. Ammonium pirate glycerine was applied to very old specimens’ slides to prepare them for image acquisition. Broken and spoiled specimens were discarded during this phase.

### Image acquisition & database development

In this study, only the diagnostic parts of monogeneans such as anchors, bars and copulatory organs were considered for digitisation. In some slides soft parts of specimens were damaged due to pressure of sliding during preparation of slides and this resulted to a very messy background for the selected organs which are the hard parts. Such slides were avoided in digitization as the hard parts were not easy to segment.

The quality of images depends on the model of microscope, lenses and camera specifications. The resolution of the captured images was 1044 × 772 pixels and all the images were saved in Tagged Image File format (TIF). All acquired images were indexed according to slide tags. A total of 102 images were taken from four species’ and the best 80 were used in this study. According to previous studies, [30, 31], we decided to use half of our digital images for training and the other half for testing the system. 10 images of each species were selected as training set and 10 were used as testing set.

### Image processing

Image processing in this study includes three essential steps: 1. Image pre-processing, 2. Image segmentation and 3. Feature extraction. Matlab R [32] was used as the Image Processing Toolbox, installed on Intel(R) Xeon (R) CPU E5-1620 v2 @ 3.70GHz, 16.00GB RAM, Windows 7 Professional (64-bit) to conduct this study.

Background feature minimization is an important pre-processing step in monogeneans classification. Otherwise, soft part features of monogeneans could mix with those from hard parts and the texture analysis will yield unreliable results. The image pre-processing follows as:

1. Images were converted to intensity images.
2. Filtering intensity images with the average correlation kernel of size 20 × 20.
3. Detecting the edge of the anchors and bars of monogeneans.

After detecting the edges in the images, image segmentation was performed where bars and anchors were identified and segmented from unwanted particles in the images:

1. The images were converted to binary images with threshold of zero. After creating average filter, we deduct the image from the filter. The result is an intensity image which contains negative and positive values. Therefore, pixels, greater than 0 will turn to 1 (white) and other pixels will turn to 0 (black).
2. Small particles (<1000 pixels) were excluded to ensure only the bars and anchors are segmented for feature extraction. Figure 2 shows the image processing steps for four species.

**Fig. 2:**
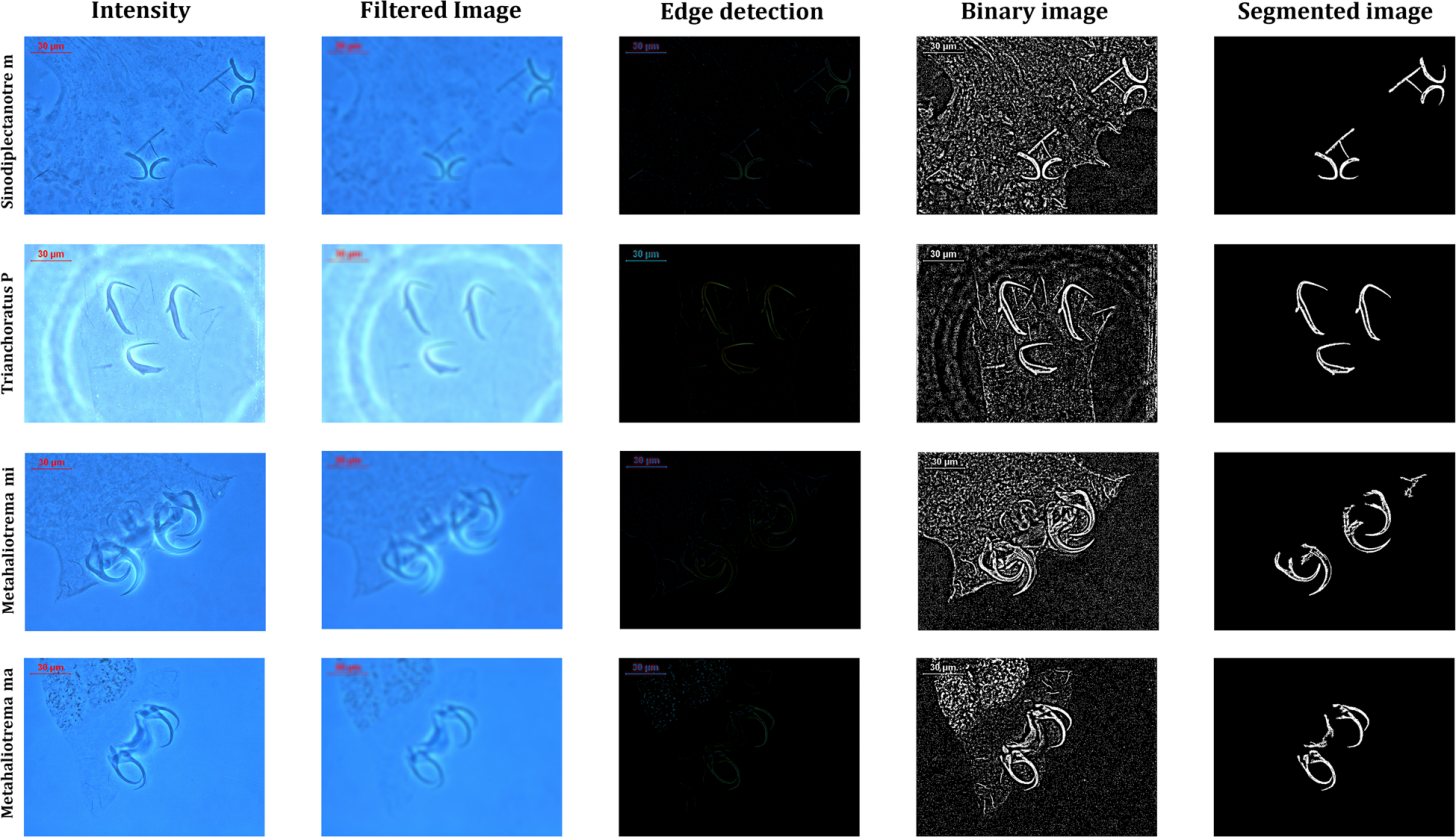
Process in image pre-processing, edge detection and image segmentation steps for four species of *Sinodiplectanotrema malayanus, Trianchoratus pahangensis, Metahaliotrema mizellei* and *Metahaliotrema sp.*
3. Features were extracted from the shape descriptors represented by the binary images of the bars and anchors, using appropriate functions in Matlab. The 10 features extracted were: Euler number, perimeter, area, area density, perimeter density, centre of bounding box, length of bounding box, width of bounding box and orientation of bounding box.

### Feature selection

To increase the performance of KNN and decrease the number of unnecessary features, Linear Discriminant Analysis (LDA) was applied for feature selection. Practically, LDA as a feature dimensionality reduction technique would be a pre-step for a typical classification task. In LDA, first the d-dimensional mean vectors and scatter matrices were computed. Next, to obtain the linear discriminants the generalized eigenvalue problem was solved. Then linear discriminants for the new feature subspace was selected and finally the samples were transferred onto the new subspace. The eigenvector corresponding to a larger eigenvalue is more efficient in capturing discriminative information of the sample [33].

### Classification

We applied K-nearest neighbour (KNN) classifier to the same training and test datasets. K-NN, as a non-parametric classifier, identifies the test sample by a majority vote of its neighbours which are assigned to the class that is most common among its K nearest neighbours. The KNN parameter was set to 10 in this study. The three selected features obtained from the previous stage were used as input to the KNN classifier. Four species of monogeneans were used and the vectors of image labels were prepared according to their features. Since the sample size is small in this study we applied Leave-One-Out (LOO) and 10 fold cross validation to assess how the results of our system generalize to an independent data set.

## Results

### Feature selection

**Fig. 3:**
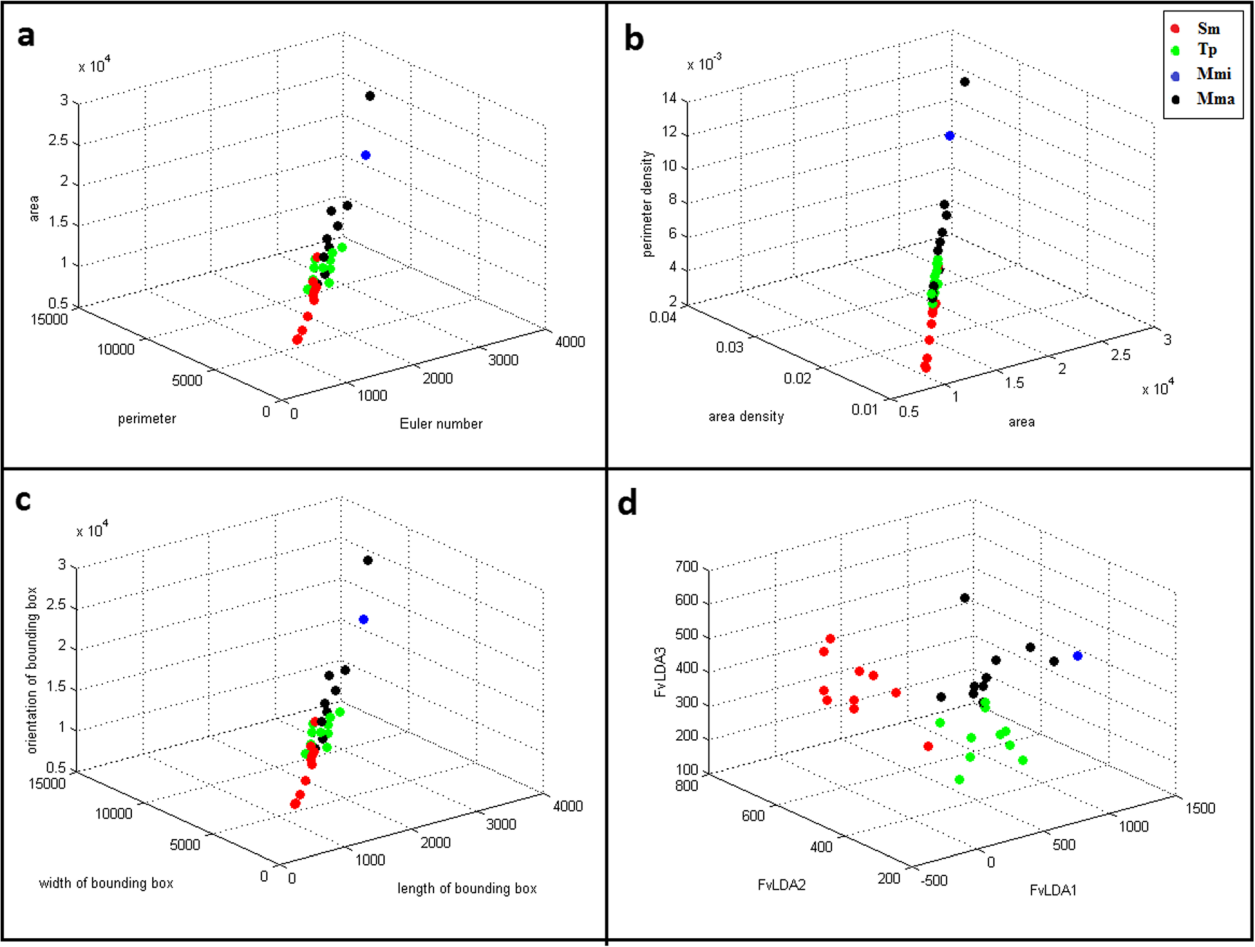
3D scatter plot with different features. **a** scatter plot with combination of three features which are Euler number, perimeter, area (**b**) scatter plot with combination of three features which are, area, area density, perimeter density (**c**) scatter plot with combination of three features which are length of bounding box, width of bounding box and orientation of bounding box (**d**) scatter plot with combination of LDA selected features: FvLDA1, FvLDA2 and FvLDA3. The data were classified into 4 species: *Sinodiplectanotrema malayanus (Sm), Trianchoratus pahangensis (Tp), Metahaliotrema mizellei (Mmi)* and *Metahaliotrema sp. (Mma)*

As a result of LDA, three embedding functions were calculated for the feature vectors. Three features were selected for training the K-nearest neighbour classifier. The feature selection method increased the classification results from 75 to 90%. Figure 3 demonstrates 3D scatter plots of the classification result for 4 species with 10 extracted features and three features after LDA.

### K-nearest neighbour (KNN) Training

KNN does not make any hypothesis on the underlying data distribution. This is pretty useful in our case as the data is from the real world. Generally practical data does not follow the theoretical assumptions like for example Gaussian mixtures or linearly separable. Non parametric algorithms like KNN come to the rescue here. The trained model was constructed using 10 images of each monogenean species. Basically we tried to find the k nearest neighbour and do a majority voting. The best result with *k* = 10 nearest neighbours was 90%.

### System evaluation

Images of four species of monogeneans namely *Sinodiplectanotrema malayanus, Trianchoratus pahangensis, Metahaliotrema mizellei* and *Metahaliotrema* sp. were used in this study. The performance of the system was evaluated by comparing the output from the trained model with the predicted result using the testing dataset as the input. The testing dataset of monogenean images was a new independent dataset not used for the training. 10 images of each species were used for testing the system. The accuracy is calculated according to the confusion matrix of the testing dataset. This means the sum of correct prediction, divided by number of all data. Based on our results, the technique presented in this study was able to recognise and identify most of the monogeneans correctly with an overall accuracy of 90%. All *Metahaliotrema mizellei* specimens were identified correctly; one specimen from *Sinodiplectanotrema malayanus* was misidentified as *Trianchoratus pahangensis,* two specimens of *Trianchoratus pahangensis* were misidentified as *Metahaliotrema* sp. and one specimen from *Metahaliotrema* sp. was misidentified as *Trianchoratus pahangensis*. Confusion matrix (Table 1) demonstrates the classification result.

**Table 1:** Confusion matrix of testing dataset used for system evaluation

Species		Results			Accuracy %
	Sm	Tp	Mmi	Mma	
Sm	9	1	0	0	90
Tp	0	8	0	2	80
Mmi	0	0	10	0	100
Mma	0	1	0	9	90
Overall					90

The confusion matrices showing the classification of 4 species of Monogeneans with *k* = 10

The data was classified into 4 species: *Sinodiplectanotrema malayanus (Sm), Trianchoratus pahangensis (Tp), Metahaliotrema mizellei (Mmi)* and *Metahaliotrema sp. (Mma)*

## Discussions

An automatic identification system for monogenean species is proposed in this study. The system was trained using 40 images of four species and tested by another 40 images of the same species. A total of 102 images were captured and digitised, however 22 were eliminated due to noise and overlapping of anchors and bars that might lead to misclassification [34, 35]. All captured images were stored in an image database for easy retrieval by the classifier to train and test the system. K-nearest neighbour was used for detection of four species of monogeneans according to their diagnostic parts. The accuracy of automated identification in this study is 90%. Features such as Euler number, perimeter, area, area density, perimeter density, centre of bounding box, length of bounding box, width of bounding box and orientation of bounding box were extracted from anchors and bars of monogeneans. The overlapping of bars and anchors of most sample images prevented good feature extraction. Using LDA for feature selection, a new feature vector with 3 features was established. The selected features were given to KNN and as result *Metahaliotrema mizellei* specimens were identified correctly. Mainly anchor and bar size in this species were totally distinct; one specimen from *Sinodiplectanotrema malayanus* and also one specimen from *Metahaliotrema* sp. were misidentified as *Trianchoratus pahangensis.* The shape of anchor organs’ tail in *Sinodiplectanotrema malayanus* species and size of anchors in *Metahaliotrema* sp. specimens are similar to *Trianchoratus pahangensis* specimens. Two specimens of *Trianchoratus pahangensis* were misidentified as *Metahaliotrema* sp. The anchors of these two species are very similar in size and shape of their tails. The features are very much dependent on the size and shape. In the future, different image processing techniques will be adopted to differentiate these two species. In some images anchors were separated and in some they were overlapping with other anchors and bars, therefore, this causes turbulence in feature extraction.

We used the images to calculate the LOO and 10 fold cross validation. The error rate from LOO cross validation is 0.1 and the accuracy is 91.25%. We also divided all of the digital images to 10 subset, one subset was held out as training and the other sets were held out as testing set. The error rate resulted for 10 fold cross validation is this study is 0.1 and accuracy is 92.5%. The results from LOO and 10 fold cross validation are better than KNN classification. (Tables 1, 2 and 3).

**Table 2:** Confusion matrix of leave one out cross validation

Species		Results			Accuracy %
	Sm	Tp	Mmi	Mma	
Sm	19	1	0	0	95
Tp	0	18	0	2	90
Mmi	0	0	20	0	100
Mma	0	4	0	16	80
Overall					91.25

The data was classified into 4 species: *Sinodiplectanotrema malayanus (Sm), Trianchoratus pahangensis (Tp), Metahaliotrema mizellei (Mmi)* and *Metahaliotrema sp. (Mma)*

**Table 3:** Confusion matrix of 10 fold cross validation

Species		Results			Accuracy %
	Sm	Tp	Mmi	Mma	
Sm	19	0	1	0	95
Tp	0	18	2	0	90
Mmi	0	0	20	0	100
Mma	0	3	0	17	85
Overall					92. 5

The data was classified into 4 species: *Sinodiplectanotrema malayanus (Sm), Trianchoratus pahangensis (Tp), Metahaliotrema mizellei (Mmi)* and *Metahaliotrema sp. (Mma)*

In this study, only bars and anchors of monogeneans were used as diagnostic organs for species identification. In future studies marginal hooks and copulatory organs can be considered. Improving the image quality could also yield better classification results.

## Conclusions

Automated identification of monogenean species based on haptoral organ images of monogeneans achieved an overall accuracy of 90%. Image processing techniques were applied to automatically extract features from microscope images followed by KNN as the classifier. An automated identification method will be useful for taxonomists and non-taxonomists since sample processing time is reduced. The enhancement of image acquisition to achieve better image quality and improvement in feature extraction techniques to accommodate large datasets covering more taxa is planned for future work. In conclusion, the purpose of this study is to develop a fully automated identification system capable of identifying monogenean specimens. Eventually, this study will be integrated into a digital biological ecosystem published by the corresponding author [36]. This work will also be extended to a species classification pipeline which incorporates semantics using textual annotations and image attributes.

## Abbreviations

ABIS: Automated bee identification system
AIICHLA: Automated insect identification through concatenated histograms of local appearance
AIMS: Automatic identification and characterization of microbial populations
ALIS: Automated leafhopper identification system
ASM: Active shape models
DAISY: Digital automated identification system
DiCANN: Dinoflagellate categorization
KNN: K-nearest neighbour
LDA: Linear discriminant analysis
LOO: Leave one out
MLP: Multilayer perceptron
SPIDA: Species identification, automated
SVM: Support vector machine
TIF: Tagged image file format

## References

[1] Cesar A, Jane S, Sandra F, Arthur G, Luciano DF (2006). Biological shape characterization for automatic image recognition and diagnosis of protozoan parasites of the genus Eimeria. Pattern Recogn Soc.

[2] Brooks D, Mclennan D (1993). Comparative study of adaptive radiations with an example using parasitic flatworms. Am Nat.

[3] Whittington ID (1998). Diversity “down under”: monogeneans in the Antipodes (Australia) with a prediction of monogenean biodiversity worldwide. Int J Parasitol.

[4] Kearn GC (1994). Evolutionary expansion of the Monogenea. Int J Parasitol.

[5] Boeger WA, Kritsky DC (1993). Phylogeny and a revised classification of the Monogenoidea Bychowsky. Syst Parasitol.

[6] Vignon M (2011). Putting in shape – towards a unified approach for the taxonomic description of monogenean haptoral hard parts. Syst Parasitol.

[7] Vignon M (2011). Inference in morphological taxonomy using collinear data and small sample sizes: Monogenean sclerites (Platyhelminthes) as a case study. Zool Scr.

[8] Martins J, Oliveira LS, Nisgoski S, Sabourin R (2013). A database for automatic classification of forest species. Mach Vision Appl.

[9] Coltelli P, Barsanti L, Evangelista V, Frassanito AM, Gualtieri P (2014). Water monitoring: automated and real time identification and classification of algae using digital microscopy. Environ Sci Process Impacts.

[10] Sigal T, Hayit G, Gabi T, Shimon A (2004). Automatic identification of bacterial types using statistical imaging methods. IEEE Trans Med Imaging.

[11] Kevin JG, Mark AO (2004). Automated species identification: why not?. Philos T R Soc B.

[12] Kiranyaz S, Ince T, Pulkkinen J, Gabbouj M, Ärje J, Kärkkäinen S, Tirronen V, Juhola M, Turpeinen T, Meissner K (2011). Classification and retrieval on macroinvertebrate image databases. Comput Biol Med.

[13] Natalia L, Hongli D, Wei Z, Matt S, Jenny Y, Robert P, Andrew M, David AL, Salvador RC, Eric NM, Linda GS, Thomas GD (2008). Automated insect identification through concatenated histograms of local appearance features: feature vector generation and region detection for deformable objects. Mach Vision Appl.

[14] Guru DS, Sharath YH, Manjunath S (2010). Texture Features and KNN in Classification of Flower Images. IJCA Special Issue on “Recent Trends in Image Processing and Pattern Recognition”. RTIPPR.

[15] Rozniza A, Amir H, James EB, Andrew PS. The use of ASM feature extraction and machine learning for the discrimination of members of the fish ectoparasite genus gyrodactylus. Paper presented at the Neural Information Processing, 2012; 7666: 256–263.

[16] Culverhouse PF, Simpson RG, Ellis R, Lindley JA, Williams R, Parisini T, Reguera B, Bravo I, Zoppoli R, Earnshaw G, McCall H, Smith G (1996). Automatic classification of field-collected dinoflagellates by artificial neural network. Mar Ecol Prog Ser.

[17] Thamsanqa M, Shaun B, Greg F. The identification of mammalian species through the classification of hair patterns using image pattern recognition. Proceedings of the 4th international conference on Computer graphics, virtual reality, visualisation and interaction in Africa, 2006; 177–181.

[18] Guannan Y, Nils H, James R, Reinhard K, Bodo R. Understanding Tracks of Different Species of Rats. Communication and Information Technology Research Technical Report 187, 2006

[19] Christopher HD, Christopher DP (1994). Automated identification of leafhoppers (Homoptera: Cicadellidae: Draeculacephala Ball). Ann Entomol Soc Am.

[20] Morgan EO, Gauld ID, Kevin JG, Weeks P. Daisy: an automated invertebrate identification system using holistic vision techniques. in Proceedings of the Inaugural Meeting BioNET-INTERNATIONAL Group for Computer-Aided Taxonomy (BIGCAT), 1997; 13–22.

[21] Richard J, René G, Glen T, Linda M, Malcolm W, Laura G, Laura Z, Lynne B (2000). Automated identification and characterisation of microbial populations using flow cytometry: the AIMS project. Sci Mar.

[22] Tom A, Stefan S, Volker S, Dieter W, Biodiversity informatics in action: identification and monitoring of bee species using ABIS. Paper presented at the Proc. 15th Int. Symp. Informatics for Environmental Protection, 2001; 425 – 430.

[23] Hanqing Z, Zuorui S. On computer-aided insect identification through math-morphology features. J China Agric Univ. 2002;7:38–42.

[24] Jongman C, Junghyeon C, Mu Q, Chang W, Hwang YK, Ki B, Tae SC (2007). Automatic identification of whiteflies, aphids and thrips in greenhouse based on image analysis. Math Comput Simulat.

[25] Kimberly NR, Martin TD, Jeremy CH, Norman IP (2007). Introducing SPIDA-web: wavelets, neural networks and Internet accessibility in an image-based automated identification system. Syst Assoc Spec Vol.

[26] Liu F, Shen ZR, Zhang JW, Yang HZ (2008). Automatic insect identification based on color characters. Chinese Bull Entomol.

[27] Leow LK, Chew LL, Chong VC, Dhillon SK (2015). Automated identification of copepods using digital image processing and artificial neural network. BMC Bioinformatics.

[28] Abu A, Lim SLH, Sidhu AS, Dhillon SK. Biodiversity image retrieval framework for monogeneans. Syst Biodivers. 2013;11:19–33.

[29] Lim LHS, Gibson DI. Taxonomy, taxonomists & biodiversity. In: Manurung R, Zaliha CA, Fasihuddin BA & Kuek C, Eds., Biodiversity-Biotechnology: Gateway to Discoveries, Sustainable Utilization and Wealth Creation. Kuching, 2010; 33–43.

[30] Taisong J, Xueliang H, Pifan L, Feifei Z (2015). A novel method of automatic plant species identification using sparse representation of leaf tooth features. PLoS One.

[31] Mijares ST, Flores F (2016). A novel method for the separation of overlapping pollen species for automated detection and classification. Comput Math Methods Med.

[32] Image Processing Toolbox - MATLAB [Internet]. [cited 2015 Oct 19]. Available from: https://www.mathworks.com/products/image/index.html.

[33] Yang J, Jin Z, Yang J. Nonlinear Techniques for Dimension Reduction. In: Li SZ, Jain AK, editors. Encycl. Biom. [Internet]. Springer US; 2015 [cited 2016 Nov 23]. p. 1163–8. Available from: http://link.springer.com/referenceworkentry/10.1007/978-1-4899-7488-4_294.

[34] Ali R, Hussain A, Bron JE, Shinn AP. Multi-stage classification of Gyrodactylus species using machine learning and feature selection techniques. 2011 11th Int. Conf. Intell. Syst. Des. Appl. [Internet]. IEEE; 2011 [cited 2016 Sep 30]. p. 457–62. Available from: http://ieeexplore.ieee.org/document/6121698/.

[35] Strona G, Montano S, Seveso D, Galli P, Fattorini S (2013). Identification of Monogenea made easier: a new statistical procedure for an automatic selection of diagnostic linear measurements in closely related species. J Zoolog Syst Evol Res.

[36] Dhillon SK, Chiew SL, Leow LK, Sidhu AS, Shuhaimi NI, Leong YM, Chong VC (2013). A model of a digital biological ecosystem. Syst Biodivers.

